# Kirk’s Hand Book of Physiology

**Published:** 1893-11

**Authors:** 


KIRK'S HANDBOOK OF PHYSIOLOGY, by W. Morrant Baker, F. R. C. S., late Surgeon and Lecturer on Physiology at St. Bartholomeu's Hospital, etc., and Vincent Dormer Harris, M. A. Loud, F. R. C. P., Examiner in Physiology at the Coujoint Board of the Royal College of Physicians and Surgeons, and in the University of Durham; Demonstrator of Physiology at St. Bartholomeu's Hospital; Physician to the Victoria Park Hospital for Diseases of the Chest. Thirteenth edition, with upwards of five hundred illustrations, including some colored Plates. Philadelphia; P. Blackiston, Son & Co. 1892. Cloth, Demi, $8.00.

Though called a Handbook of Physiology, this edition goes far beyond the ordinary scope of a handbook. It combines the admired qualities of a handbook with the essentials of a text book, making a concise, systematically arranged text book of Physiology. It is thoroughly up to the teachings of the present time and is one of the books now recognized by the Examiners of the Royal College of Physicians and Surgeons, England, and is used by the students for their examinations for the Fellowship. It is especially adapted to the wants of the medical student, who "studies Physiology as an introduction to medicine," being,not too concise for his undertaking and not too elaborate for his limited time.

To those who are unacquainted with the book, the names of Morrant Baker and Vincent Dormer Harris as the authors are sufficient to recommend it and give it popularity. The value of this edition is greatly enhanced by the forceful and lucid style in which it is written, giving it in this respect some advantage over most books on this subject. We highly recommend it to the medical student and the busy practitioner.

V. H. T.

THE ER, A KEY TO THE U. S. P., D. O. Haynes & Co., Publishers, Detroit, Mich. Price, 25 cents.

This little book of seventy-five pages can be carried in the vest pocket, yet it contains a complete list of all the drugs and preparations in the new U. S. P, with common names and synonyms of each drug, the parts employed, the doses in both apothecaries and metric systems and the preparations in which the drug is employed. The official names are arranged alphabetically and printed in black faced type, making a handy little book for the young physician and pharmacist.

